# Is a bad character doctor much worse than a malformed one?

**DOI:** 10.1590/0100-6991e-20223048

**Published:** 2022-03-11

**Authors:** LEONARDO EMILIO DA SILVA

**Affiliations:** 1 - Faculdade de Medicina da Universidade Federal de Goiás, Departamento de Cirurgia - Goiânia - GO - Brasil

The answer is a resounding yes, with a huge exclamation point at the end. 

I could objectively close our discussion with this answer, which also carries an enormous weight of responsibility. But it would not be fair to most doctors, beautiful in character, who dedicate almost all their lives to lives of others.

Recently, amidst the restless and ordinary daily bombardment of messages on my social media videos, messages, “Good morning” - what I believe is the routine of the billions of quarantined humans on this planet - I had to stop and sit down!

I was affected by a series of videos, very rich in hideousness, of a doctor exposing her patients and their surgical procedures amid dancing, faces and mouths filled with little music, all poorly edited with tasteless refinements, in one of these applications that allow the instant creation of startup investors millionaires, in addition to sterile celebrities and, in this case, dangerous for the medical community and society.

You could ask me: what’s the danger, what’s the problem in editing and posting videos with little dances, little songs, and many other “littles”, a fact so common in all social networks (littles)?

The answer would be no problem in a huge social media environment. But we are talking - for those who have not seen the videos (I beg you, please continue without watching them!) - of a doctor exposing human body parts (surgical specimens) without any shame, almost like juggling in a circus of horrors. I’m not going to stick to the limits of social networks, it would be the subject of another article and, by the way, an endless “tail chasing”.

It is important to emphasize, with a teaching view, the limits imposed by the laws in force in the country. The Brazilian Constitution recognizes the fundamental rights with the objective of protecting the essential dignity of the human person, both in the individual and social perspective. The Constitution elevates the principle of dignity to the hierarchy of rule number one of fundamental rights (right to life; to physical integrity; to liberty; to equality; to honor).

In this perspective of fundamental rights, the Code of Medical Ethics (CEM)^1^ contained in CFM Resolution No. 2217, of September 27, 2018 (modified by CFM Resolutions No. 2.222/2018 and 2.226/2019), constituted by its eleven fundamental rights and 117 duties of Brazilian physicians, is unequivocal:

II - The aim of the physician’s attention is the health of the human being, for the benefit of which he/she must act with the utmost zeal and the best of his/her professional capacity.
IV - The physician is responsible for ensuring and working for the perfect ethical performance of medicine, as well as for the prestige and good reputation of the profession.
V - It is up to the physicians to continuously improve their knowledge and use the best of scientific progress for the benefit of patients and society.
VI - The physician will have absolute respect for the human beings and will always act in their benefit, even after death. [...]
XI - The physician will keep confidential the information that he/she has knowledge of in the performance of his/her duties, except for cases provided for by law.

Well, after multiple posts by the medical lady, the Brazilian Society of Plastic Surgery was very effective and quickly suspended her from its membership. Then, the Regional Council of Medicine (CREMESP) interdicted her as a precaution, within the legal framework - namely, the CEM in its general provisions, paragraph II, “Physicians who commit serious faults provided for in this Code and whose continued professional practice constitutes a risk of irreparable damage to the patient or society may have their professional practice suspended through a specific administrative procedure”.

To the surprise of some, after her interdiction was made official by CREMESP, the doctor made many videos confronting the measure, referring to the family tragedy of the past that made her unshakeable, almost a character in the struggle for a large bank account. Soon, there were comments that she was a person with some (or all) degree(s) of psychopathy - I even believe that many medical commentators had personal interests in defending her bad character. And that’s the heart of my narrative: Does a mental illness make someone a bad character?

Of course not, and the main responsible for this association is the uncomplex, linear argumentation that associates any “bad thing” as being mental illness and converting sanity into something positive. Human history is full of similar examples, very dangerous people without any psychiatric problem, and vice versa. Herein lies the great ambiguity of this linear thinking, this tendency to summarize facts that we do not understand clearly so that they make sense, which in most cases corresponds to quack reality.

Being a carrier of psychopathy does not imply being bad. On the other hand, character (oh, character...), one of the components of our personality, in the philosophical definition, the bad character, often with a specialist title veil or occupying class honorific positions without the slightest honor and character, acts against the nature of reason, becoming responsibly incapable of perceiving the hierarchy of values that should guide human actions. The bad character suffers, therefore, from a very serious cognitive deficit that, when discovered, calls for divine goodness, almost a parallel with using God´s name in vain (they use hashtags involving God, Jesus, the Creator... but deliberately and continuously disobey His commandments). 

Here, I will refer to the set of Saint Thomas Aquinas’ work, who states that when it is said that a person has the power to do something, we are dealing with what the soul can do (its faculties). According to him, the will and the intellect (higher faculties of the human soul) are affected in individuals of bad character. And so, one’s repeated acts moved by pride, vainglory, cupidity... make one deprive oneself of the greatest virtue in the practical sphere of reason: prudence, defined by Saint Thomas Aquinas as the “right reason in acting”^1^.

In this scenario, already answering the initial question, a bad character doctor is indeed MUCH worse than a malformed one. The malformed, good character one follows the Hippocratic oath, and modesty from the cradle, in addition to always seeking help (from knowledge or from other more experienced colleagues). The bad character, on the other hand, is filled with ethical lapses that usually involve short-term goals, without the slightest concern for the patient, only with his exhibitionist vainglory guided by the seven deadly sins that vary in intensity, depending on the audience.

As I said, these are driven by the farcical need for a sterile amount of people with psychiatric disorders (or not?), faithful followers and repeaters of “likes”, “shares”, “saves”, who end up being victims of their own “touch screen clicks” in the audience of a bad character doctor, with a specialist title, who clearly uses them to fatten his/her bank accounts at the expense of crimes elicited by sheer greed. The profile is almost always the same, they sell the (very expensive) concept of a Medicine that is always happy and free from complications and shame, until there is an urgent demand for a trained doctor who is faithful to the Hippocratic oath to resolve the always tragic complications, some of which are even very serious aberrations. This colleague will save a life with respect and modesty above all, even not denouncing these bad characters. Once the crime takes place, whatever it may be, these evil characters become, in front of not always exempt eyes, people with “some psychiatric disorder”, “God-fearing”, “disproportionately punished”... Enough! Please respect my insanity and my character, which I believe is good (within my sanity). Finally, the pathologically insane should receive adequate and prompt treatment; the bad characters, the rigor of the law, so that we don’t go insane! And paraphrasing Sidney Silveira in his Aquinas style: “let us not be mistaken: by losing the right reason in acting, the bad character also lost the right reason in thinking”^2^.
